# Decoding chirality at the nanoscale with momentum-space polarimetry

**DOI:** 10.1038/s41377-026-02336-z

**Published:** 2026-05-18

**Authors:** Jeeban Kumar Nayak, Meghna Sarkar, Siarhei Zavatski, Ebru Buhara, Sergejs Boroviks, Olivier J. F. Martin

**Affiliations:** Nanophotonics and Metrology Laboratory (NAM), Swiss Federal Institute of Technology Lausanne (EPFL), Lausanne, Switzerland

**Keywords:** Metamaterials, Imaging and sensing, Nanophotonics and plasmonics

## Abstract

Engineering optical chirality at the nanoscale has unlocked a wide range of light-matter interactions, with implications for the controlled manipulation of photonic degrees of freedom, ultrasensitive enantiomer detection, structured illumination microscopy, and quantum communication. Efficient characterization of chiral nanostructures is therefore of paramount importance, as it provides direct insights into their chiro-optical responses and guides the rational design of next-generation nanodevices. Conventional chiro-optical techniques, however, often fall short due to intrinsic limitations, such as their inability to probe spatially and angularly inhomogeneous chirality or to disentangle coexisting linear and circular anisotropies. Here, we present a Fourier-domain polarimetric framework to investigate the chiro-optical responses of plasmonic gammadion nanoarrays. By mapping scattered polarization states in momentum space through Stokes-Mueller polarimetry, we capture inhomogeneous radiation patterns that encode the underlying electromagnetic modes and diffraction features governing the observed chirality within the nanostructured system. The momentum-resolved Mueller matrix not only enables simultaneous quantification of circular birefringence and circular diattenuation but also facilitates their decoupling from linear anisotropies, thereby providing a comprehensive characterization of intrinsic chiro-optical behavior. We further show how structural thickness modulates the chiral response and demonstrate the sensitivity of this approach in detecting subtle chiro-optical signals. Finally, we combine gammadions arrays with momentum-domain chiral measurements as a sensitive platform for molecular enantiomer detection, opening new opportunities for advanced chiral sensing applications.

## Abstract (web summary)

Schematic illustration of Fourier-domain Mueller matrix polarimetry probing light-matter interactions in gammadion nanostructures, and enabling extraction of intrinsic chiral response for molecular enantiomer sensing.

## Graphical abstract

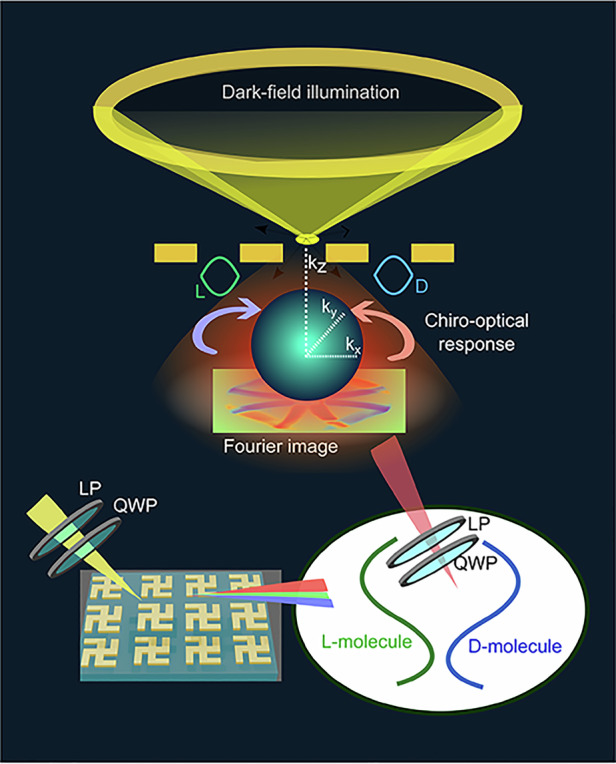

## Introduction

A chiral medium intrinsically couples the electric and magnetic fields, causing its optical properties to depend on the polarization helicity of the incident electromagnetic wave^1,2^. Consequently, right- and left-handed circularly polarized (RCP and LCP) lights experience different complex refractive indices while propagating through such a medium. A difference in the real part of these indices leads to opposite phase accumulation for RCP and LCP, manifesting itself as the rotation of an incoming linearly polarized light, commonly known as circular birefringence (CB) or optical rotation. On the other hand, a difference in the imaginary part of the refractive indices results in unequal absorption of the two circular polarization states, termed circular dichroism (CD)^1,2^. A chiral object, together with its non-superimposable mirror image, the enantiomers, exhibits opposite CB and CD responses, making these observables essential tools for characterizing a chiral medium or object^3–6^.

Chirality is ubiquitous in nature, particularly among biomolecules. The handedness of such molecules can drastically influence their physical, chemical, and biological functions. For instance, one enantiomer of a compound may serve as a therapeutic drug, while its mirror image can be toxic. Accurately distinguishing between enantiomers is therefore crucial in various fields such as molecular chemistry^7^, drug development^8^, and biomedical diagnostics^9,10^. However, naturally occurring chiral media typically exhibit very weak chiro-optical signals, making it challenging to probe and distinguish the handedness using conventional chiro-optical characterization techniques (CD and CB spectroscopy)^10–12^.

In contrast, artificially engineered media composed of 2D and 3D nanostructures can exhibit strongly enhanced chiro-optical responses^11,13–15^. Such enhancement often owes its origin to geometrical asymmetry combined with plasmonic^16,17^ and photonic resonances^2^, higher-order electromagnetic modes^18,19^, and local field enhancement from resonance coupling and hybridization^20^. These chiral nanostructures with significantly enhanced chirality can serve as highly sensitive platforms for molecular enantiomer detection^5,17,21,22^. In these systems, the strong and localized chiral near fields generated by the nanostructures interact differently with opposite molecular enantiomers, enabling label-free and highly selective sensing^22,23^. Additionally, chiral nanostructures offer control over multiple photonic degrees of freedom, permitting applications in optical manipulation^24^, holographic encryption^25^, structured illumination microscopy^26^, chiral lasing^27^, and quantum communication^28^.

The rapid advances in chiral nanophotonics demand experimental approaches capable of efficiently probing the complex optical responses of nanostructured systems, which remains challenging in practice^19^. For instance, a natural chiral object often exhibits simultaneously both linear and circular anisotrpic effects^29^. While circular anisotropies such as CB and CD arise from the helicity-dependent refractive indices, linear anisotropy originates due to the different refractive indices sensed by two orthogonal linearly polarized states. Under circularly polarized illumination, both the ellipticity and orientation angle of the output polarized state are influenced by the coexistence of linear and circular anisotrpy within the system. As a result, although these anisotropies originate from distinct physical mechanisms, they often become intertwined in standard ellipsometric measurements, complicating the reliable quantification of chirality. Moreover, several chiral nanostructures exploit the excitation of higher-order multipoles, and coupled plasmonic-photonic resonances to achieve giant chirality. Such structures are expected to simultaneously exhibit multiple polarization effects, including linear and circular birefringence, linear and circular diattenuation, as well as depolarization, which cannot be readily disentangled or accurately analyzed using standard chiro-optical measurement techniques. Accurate decoupling of linear and circular anisotropy is thus essential for the reliable extraction of the true chiro-optical response of nanostructures, a critical metric in chiral molecular sensing^30,31^, and has gained significant recent attention^31,32^.

Meanwhile, conventional CD and CB (optical rotatory dispersion) spectroscopy, provide only the spectral dependence of chiro-optical signals, without resolving their spatial or angular distributions. Such an omission can obscure crucial information on the light-matter interactions within nanostructured systems. Indeed, chiral nanostructures, often exhibit inhomogeneous or angular-dependent radiation patterns that encode the underlying multipolar electromagnetic modes and other resonances excited within the structure and their contributions to the resultant chirality^19,32^.

Fourier-plane imaging offers a powerful route to address these challenges. By capturing direction-dependent polarized radiation patterns in momentum space, Fourier domain measurements provides access to light-matter interactions that remain hidden in conventional CD measurements^33^. In particular, momentum-domain (*k*-space) polarimetry enables the exploration of a broader range of phenomena in chiral nanostructures, including spin-orbit coupling induced by symmetry breaking^34^, spin-dependent diffraction, and handedness-selective orbital angular momentum generation^35^. However, most existing polarization-resolved momentum-space imaging approaches probe only selected Stokes parameters by relying on linear or circular polarization contrasts. As a result, they often fail to fully characterize polarization conversion, depolarization, disentanglement between linear-circular anisotropies effects that are particularly important in chiral nanostructures where multiple electromagnetic resonances coexist. Accordingly, angular-dependent polarization conversion in chiral metasurfaces is still not properly understood^19,36^. Retrieving complete polarization maps across momentum space is of particular interest as it enables the discovery and analysis of spatially structured polarization fields generated by chiral metasurfaces—an aspect that remains largely unexplored in chiral nanophotonics. These capabilities can significantly extend the scope of chiral nanophotonics by establishing nanostructures as versatile building blocks for multifunctional photonic devices. Therefore, there is a clear need for advanced characterization techniques capable of providing a comprehensive characterization of the polarized light matter interactions in chiral metasurfaces.

In this work, we introduce a Fourier-domain Mueller matrix polarimetry platform to investigate chiro-optical effects in plasmonic gammadion nanoarrays. Implemented in a dark-field configuration, the momentum-resolved Mueller matrix measurements provide a complete and quantitative description of the far-field, direction-dependent, polarization response exclusively associated with the chiral metasurfaces. The polarization states of the scattered light are mapped across spatial frequencies within the Stokes-Mueller formalism, enabling direct access to structured polarization responses generated by the nanostructured system. The momentum-space Mueller matrix simultaneously reveals polarization conversions, depolarization behavior, and mixed linear-circular anisotropies exhibited by the nanostructures. This multidimensional information allows unambiguous separation between the linear and circular anisotropies, leading to the extraction and quantification of the true CB, and circular diattenuation (CDA) contributions generated by the gammadion arrays. This unique ability is then exploited to detect and quantify weak chiro-optical responses from thin gammadion structures. Moreover, by resolving the angular dependence of the chiro-optical parameters, our frameworks provide physical insights into the mutual coupling between the electromagnetic modes excited in the structure, their coupling to the far-filed and their polarization topology. Finally, the extracted intrinsic chiro-optical responses of the gammadion arrays are employed as a direct metric for chiral molecular sensing, establishing momentum-resolved Mueller matrix polarimetry as an efficient and versatile platform for enantiomer discrimination and opening new avenues for advanced chiral sensing and nanophotonic applications.

## Results

**Fig. 1:**
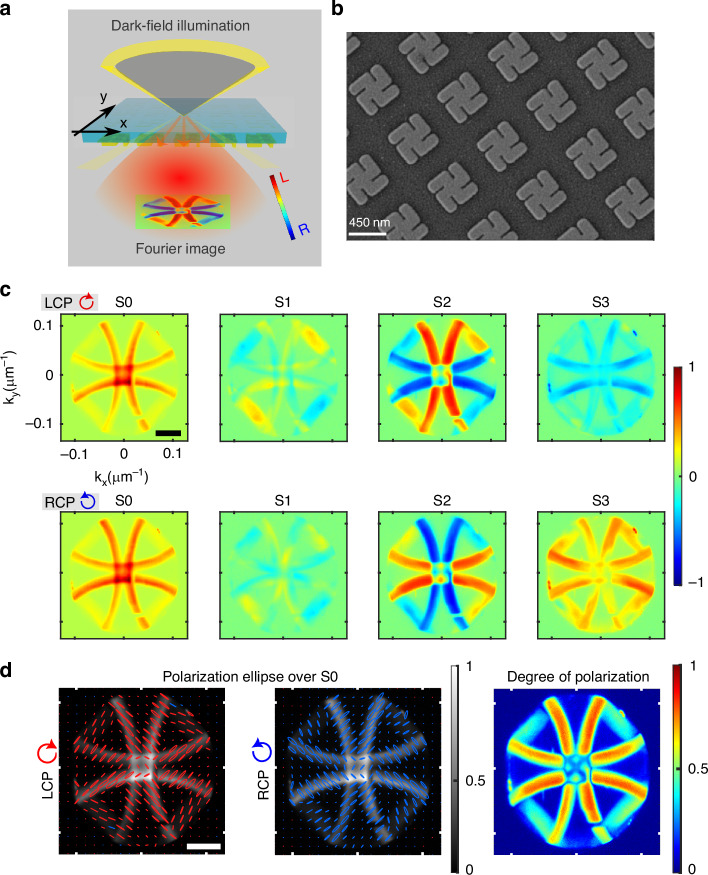
Momentum domain polarization response of the plasmonic gammadion array. **a** Schematic illustration of the dark-field illumination geometry and the resulting inhomogeneous polarization distribution observed in the scattered intensity pattern from the gammadion array. **b** Scanning electron microscopy image of a typical fabricated array. **c** Momentum-space maps of the Stokes parameters from right-handed gammadion arrays under LCP and RCP illuminations, highlighting the polarization-dependent scattering features. **d** Distribution of the polarization ellipses and the corresponding degree of polarization in the Fourier space. The scale bar corresponds to 0.05 μm^−^^1^

Our experimental configuration Fig. 1a, integrates Fourier imaging with polarimetry to capture the angular distribution of the polarization-resolved scattering pattern from the nanostructured gammadion arrays. A custom-built inverted microscope (Olympus IX73) is utilized for broadband excitation (400–1000 nm) and the subsequent collection of scattered light, using a microscope objective (Olympus, 50x magnification, numerical aperture NA = 0.5). Investigating light-matter interactions in nanostructures via Fourier microscopy often suffers from the intrinsic weak scattered signal associated with the sample. Furthermore, in this configuration the collected signal is spread across the spatial frequencies, making it difficult to interpret the angular scattering patterns^37^.

To overcome these challenges, we employ a periodic array of nanostructures Fig. 1b and adopt a dark-field illumination configuration, which ensures the collection of scattered light exclusively from the nanoarray by eliminating the unwanted incident background signal. The use of a periodic gammadion array offers several advantages by providing a high signal-to-noise ratio with well-defined diffraction features, making it efficient for reliable Mueller-matrix measurements in momentum space. Additionally, periodic arrays generate uniformly distributed chiral near-field hot-spots, providing spatially averaged enhancement for chiral molecular sensing. The back focal plane of the objective, containing the angularly resolved scattering pattern, is projected onto a spectrometer (Andor electron multiplying charge coupled device, Kymera 328i) using an additional Fourier lens. A pair of linear polarizers and quarter-wave plates is placed in both the illumination and detection paths to record polarization-resolved radiation patterns. Polarization filters in the illumination path generate specific input polarization states, while a second pair in the detection path analyzes the polarization state of the scattered light. By systematically varying the input and output polarization states, we record the desired polarization-resolved radiation patterns from the plasmonic gammadion arrays^38^.

The geometrical parameters of the gold gammadion nanoarrays (periodicity, arm length, and thickness) were optimized via numerical simulations. A scanning electron microscopy (SEM) image of a typical fabricated array is shown in Fig. 1b, see “Materials and methods” for details of the fabrication procedure. The structures are designed to exhibit chiro-optical responses within the operating wavelength range of 600–700 nm. Both simulations and experimentally obtained CD spectra from the gammadion structures reveal a similar trend (see Supplementary Information Section S1), albeit on a narrower wavelength range. Based on these observations, a working wavelength range of 649–709 nm is selected for the experiments, and the Fourier images are captured accordingly. This spectral window is isolated using a band-pass filter in the detection path.

The intensity pattern in the Fourier plane appears as closely spaced bright arcs or semi-rings diffracted along both the *x*-, and *y*-directions, reflecting the two-dimensional (2D) periodicity of the arrays. These intensity maps carry crucial information regarding the coupling and hybridization between the electromagnetic modes excited within the structure, and the subsequent far-field optical effects are manifested on top of these intensity patterns. In the Supplementary Information Section S2 we provide schematic diagrams illustrating the appearance of ring-like patterns in the Fourier plane. Depending on the numerical aperture of the objective, only segments of these rings are captured, leading to arc-like intensity distributions.

The optical responses of a specific handedness gammadion structures are expected to differ for LCP and RCP illuminations. Accordingly, we begin by recording the momentum *k*-domain Stokes parameters with incident LCP and RCP excitations for right handed gammadion arrays, Fig. 1c. The Stokes parameters are obtained by recording the polarization projection intensities of the scattered light with six standard polarization states (0°(*I*_*H*_), 45°(*I*_*P*_), 90°(*I*_*V*_), 135°(*I*_*M*_), *LCP*(*I*_*L*_), *RCP*(*I*_*R*_)), and the Stokes parameters are defined as:

1 $${S}_{0}={I}_{H}+{I}_{V},{S}_{1}={I}_{H}-{I}_{V},{S}_{2}={I}_{P}-{I}_{M},{S}_{3}={I}_{L}-{I}_{R}$$

The resulting Stokes maps in Fig. 1c exhibit significant spatial variations, indicating polarization inhomogeneity across the angular spectrum. Among the parameters, *S*_2_ (associated with polarization rotation), and *S*_3_ (helicity or circular polarization) show strong asymmetry between LCP and RCP excitation Fig. 1c. The *S*_2_ parameter displays a characteristic $\cos 2\phi$ lobe, likely arising from spin-orbit interactions under tightly focused dark-field illumination^34^. Meanwhile, the *S*_3_ distribution reflects helicity retention, with notable contrast between LCP and RCP illuminations. Polarization ellipses reconstructed from the Stokes parameters shown in Fig. 1d provide further insights into the inhomogeneous polarized radiation from the gammadion arrays. The orientation of the polarization across orthogonally diffracted arcs (in the *x*- and *y*-directions) appears mutually orthogonal, and reverses with the illumination helicity. The handedness of the scattered polarization ellipses, however, remains consistent with the incident polarization helicity. The output polarization states in the momentum domain remain mostly left handed with the LCP illumination, and right handed for RCP illumination.

Moreover, the degree of polarization remains high throughout the diffraction patterns for both incident LCP and RCP lights, as shown in Fig. 1d (plotted for incident RCP here). In other words the scattered light from the nanostructure is found out to be significantly polarized. It is important to note that, even under dark-field illumination, contributions from multiple scattering pathways or angular crosstalk can, in principle, spoil polarization-resolved momentum-space measurements. Identifying and excluding such contributions is therefore essential, as they may introduce unwanted noise into the polarization-resolved intensity maps. Measuring the degree of polarization provides a useful means of identifying these artifacts, since multiple scattering and angular mixing are known to produce significant depolarization. Our regions of interest in the momentum space with well-defined angular features display a high degree of polarization (close to 1, Fig. 1d), confirming that these signals arise from well-defined single-scattering pathways rather than composite or mixed contributions. To probe the complete polarized light-matter interaction associated with the gammadion arrays, the 4 × 4 Mueller matrix in the Fourier plane is recorded next.

Before diving into that, we briefly discuss the electromagnetic modes excited in the gammadion nanostructure, their role in generating intrinsic chirality, and how their far-field signatures appear superimposed on the observed arc-like intensity patterns in the Fourier plane, as revealed by numerical simulations. Although the far-field radiation of the plasmonic array is largely dominated by diffraction orders, the excitation and near-field coupling of electromagnetic modes, particularly higher-order multipolar modes, strongly influence the polarization-dependent radiation features and constitute the physical origin of the observed chiro-optical response. This can be understood by analyzing the far-field radiation of an individual gammadion structure. For the chosen geometrical parameters, the nanostructure supports higher-order electric and magnetic multipolar resonances that are essential for strong chiro-optical effects^18^.

**Fig. 2:**
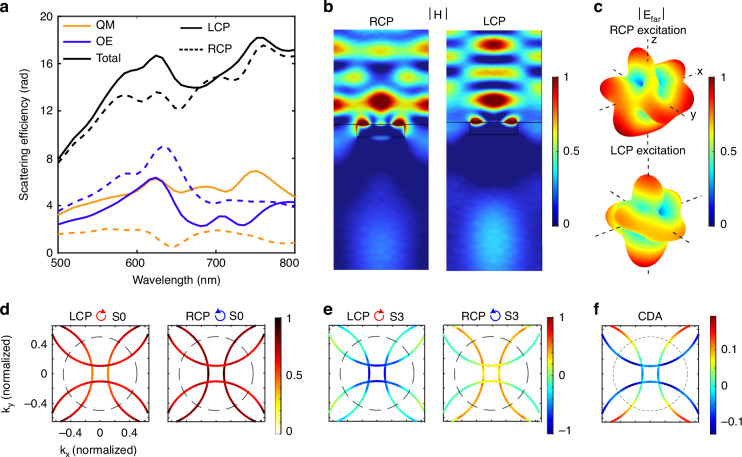
Numerical investigation of the chiro-optical response and inhomogeneous polarization scattering from gammadion nanostructures. **a** Simulated scattered efficency spectra of the gammadion structures under left-circularly polarized (LCP, black solid curve) and right-circularly polarized (RCP, black dotted curve) illuminations, shown alongside the dominant multipolar contributions: magnetic quadrupole (QM- Orange curves) and electric octupole (OE- blue curves). The solid and dotted curves represent the responses for LCP and RCP excitation. **b** Spatial distribution of the simulated total normalized magnetic field amplitude across a vertical cross section in the *y*−*z* plane for incident RCP and LCP excitations. **c** Calculated far-field electric-field radiation patterns associated with the excited electromagnetic modes of a single gammadion structure under LCP and RCP illumination. **d** Simulated scattering intensity patterns from the two-dimensional gammadion nanoarrays for opposite excitation handedness, plotted as an implicit function of the azimuthal angle or spatial frequencies. **e** Corresponding Stokes parameters *S*_3_, exhibit sign reversal for opposite circular polarization inputs. **f** Simulated momentum-domain CDA map, showing a good qualitative agreement with the experimental observations

A multipolar decomposition of the scattered field reveals dominant contributions from the electric octupole and magnetic quadrupole modes, Fig. 2a. A complete list of contributing multipoles is provided in Supplementary Information Section S3. To further elucidate the nature of these excited resonances, we analyze the spatial distribution of the magnetic field computed in the vertical cross-section (*y*−*z* plane) under different circularly polarized excitations, Fig. 2b. With incident RCP, the magnetic field features three antinodes, characteristic of an octupolar behavior; the LCP excitation results in a smaller number of antinodes, indicating the contribution from lower order multipoles. The far-field scattering patterns resulting from the excited multipolar modes are calculated at the wavelength corresponding to the maximum observed chirality, Fig. 2c. The resulting scattering profiles exhibit pronounced differences for opposite excitation handedness, thereby directly linking multipolar electromagnetic resonances to the symmetry properties of the gammadion structure and, accordingly, to the chirality exhibited by the nanostructured arrays.

The appearance of the arc-like intensity patterns in the Fourier plane, however, requires consideration of the two-dimensional periodic nature of the gammadion arrays, as these features originate from diffraction. To understand how chiro-optical signatures are manifested in these diffraction-induced arcs, we numerically investigate the far-field polarization at specific diffraction orders. In our experimental configuration, the numerical aperture of the collection objective limits detection primarily to the −1 diffraction orders. We therefore calculate the far-field polarization distribution at these orders, and the resulting intensity and polarization characteristics are summarized in Fig. 2d–f. For both left- and right-circularly polarized excitation, the simulated scattered intensity forms arc-like patterns in the Fourier plane that closely resemble the experimental observations, Fig. 2d. Superimposed on these diffraction features, the azimuthally varying Stokes parameter *S*_3_ reverses sign for opposite excitation handedness, consistent with the experimental results, Fig. 2e. Furthermore, the calculated CDA map exhibits an azimuthal variation in good qualitative agreement with the measured momentum-domain CDA maps, Fig. 2f. These simulations demonstrate that the arc-like Fourier-plane intensity patterns are dictated by array diffraction, whereas the chiro-optical modulation imprinted on these arcs originates from the differential excitation of higher-order multipoles within individual gammadion elements. We next discuss the incorporation of Mueller-matrix polarimetry to investigate the complete polarization-dependent light-matter interactions in the gammadion arrays.

**Fig. 3:**
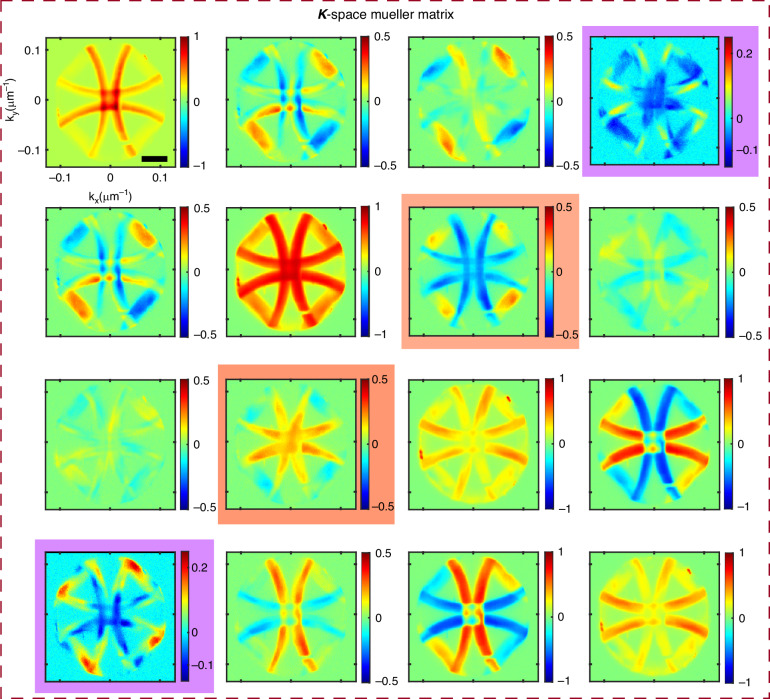
Measured Fourier domain polarization Mueller matrix corresponding to the gammadion nanoarrays. The circular anisotropy descriptor elements *M*_14_, *M*_41_ (circular diattenuation), and *M*_23_, *M*_32_ (circular birefringence) are highlighted in violet and brown colour shades. The scale bar represents 0.05 μm^−^^1^

The full 4 × 4 momentum domain polarization Mueller matrix response from right-handed gammadion arrays is recorded in the Fourier plane to enable a comprehensive characterization of the polarization-dependent light-matter interactions in the system, Fig. 3. For completeness, let us briefly summarize the physical meaning of the different Mueller matrix elements and their roles in capturing different forms of polarization anisotropy. Elements in the first row and column describe the diattenuation and polarizance effect present in the system^38,39^. Specifically, the elements *M*_12_, *M*_21_, *M*_13_, and *M*_31_ correspond to linear diattenuation, describing differential attenuation between orthogonal linear polarization states. Similarly, *M*_24_, *M*_42_, *M*_34_, and *M*_43_ represent linear birefringence, indicating phase differences between orthogonal linear polarizations^39–41^. The construction of the Mueller matrix from the polarization resolved intensities is discussed in the Supplementary Information Section S4.

Our primary focus, however, lies in the chiro-optical (or circular anisotropy) effects, which are encoded in the *M*_14_, *M*_41_, and *M*_23_, *M*_32_ elements, highlighted with colour shades in Fig. 3. The elements *M*_14_ and *M*_41_ represent CDA describing the differential absorption, scattering, or transmission between the opposite circularly polarized light (LCP and RCP). In this context, it is worth mentioning that the differential attenuation derived from the Mueller matrix offers a more generic approach compared to the conventional CD measurements^42^, which only account for the inequal absorption between the LCP and RCP; consequently, the extracted CDA parameter is incorporated throughout this work to describe the chiral responses of the gammadion array. On the other hand, *M*_23_ and *M*_32_ are associated with circular birefringence (CB), reflecting optical rotation. A hallmark signature of circular birefringence is the anti-symmetry between the *M*_23_ and *M*_32_ elements, which is observed in our experimental results (elements highlighted in brown)^39^. In contrast, linear anisotropies typically produce symmetric contributions in these elements. The circular birefringence/retardance effect can be analytically defined as *ψ* = 2*pi/λ*(*n*_*R*_ − *n*_*L*_)*t, where *n*_*L*_, *n*_*R*_ are the real parts of the refractive indices for LCP and RCP, *t* is the effective thickness of the medium, and *λ* represents the incident wavelength^1,39,41^. Owing to the presence of the CB anisotropy, rotation of the polarization orientation angle takes place for incoming linearly polarized light, which is featured in the *M*_23_ and *M*_32_ elements with a magnitude of $\sin 2\psi$ and $-\sin 2\psi$, respectively. Although the CB angle *ψ* can in principle be extracted by taking the ${\sin }^{-1}$ of these elements, we use the values of *M*_23_ and *M*_32_ directly as quantitative indicators of CB due to their relatively small magnitude in our measurements.

In contrast, the elements *M*_14_ and *M*_41_ (highlighted in violet) exhibit similar features with equal magnitude, a characteristic signature of a chiral medium. As described previously, the *M*_14_ quantifies the differential attenuation under LCP and RCP excitations, commonly referred to as the *g*-factor, or anisotropy factor of the medium^2,41^. Meanwhile, the *M*_41_ element represents the circularly polarized component of the scattered light when the incident beam is unpolarized. For a typical chiral medium, both elements yield identical values, as observed in our experimental data^39,41^. In addition to the chiro-optical effects, we also observe in Fig. 3, the signature of linear birefringence in the *M*_34_ and *M*_43_ elements of the Mueller matrix. This is likely to originate from the polarization-sensitive beam splitter present in the Olympus microscope setup^43^. In this regard, it is important to note that the recorded Mueller matrix of the sample can be influenced by the intrinsic polarization behavior of the optical elements utilized in the experimental configuration, including the illumination source, polarizer, retarders, beam splitters, and high numerical aperture condenser. Therefore, it is crucial to investigate the Mueller matrix of the system without the sample to account for these system-induced polarization effects. In our measurements, a rigorous spectral calibration procedure was implemented, and the momentum-space Mueller matrix of a blank substrate was analyzed together with the polarization alterations introduced by the high-NA dark-field condenser. This approach enables identification of the origin of individual Mueller matrix elements. The corresponding results, presented in Supplementary Information Sections S5 and S6, explicitly demonstrate that momentum-space Mueller matrix analysis effectively decouples linear and circular anisotropy effects, thereby allowing reliable extraction of the intrinsic chiro-optical response of the gammadion arrays. Additional discussion on the manifestation of chiro-optical effects, including the possible presence of asymmetric circular polarization conversion in specific Mueller matrix elements, is provided in Supplementary Information Section S4.

We next investigate the thickness-dependent chiro-optical response of the gammadion structures. The thickness of the nanostructure plays a crucial role in modulating its optical chirality, as the excited electromagnetic modes are highly sensitive to both the structure geometry and dimensions^44^. With increasing thickness, the structures not only support higher-order electromagnetic modes but also facilitate the generation of magnetic moments with non-zero components aligned with the in-plane electric dipole moment. As a result, the overall chiral response is expected to increase with thickness, which is indeed observed in our measurements, and shown in the Supplementary Information Section S1. Three different thicknesses of the gammadions, 50 nm, 100 nm, and 150 nm, with same handedness, are selected for systematic analysis. While the 50 nm thick gammadion structures exhibit a negligible CD over the entire desired wavelength range, the magnitude of the CD tends to be saturated beyond a thickness of 150 nm. This trend is consistently observed in both numerical simulations and experimental CD spectra (see Supplementary Information Section S1).

**Fig. 4:**
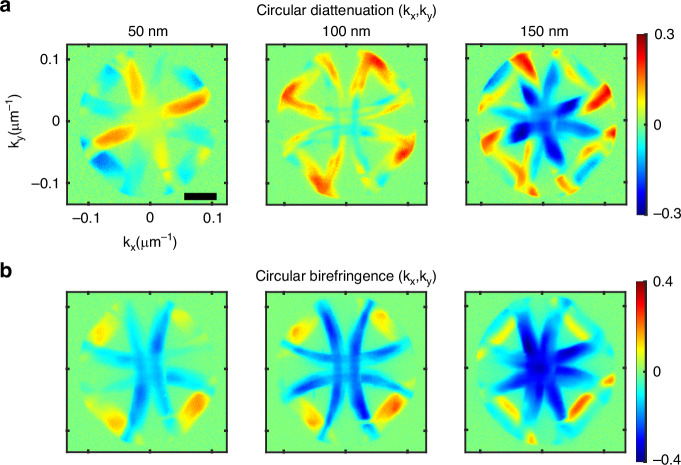
Thickness-dependent chiro-optical response of the gammadion structures. Momentum-space maps of **a** circular diattenuation and **b** circular birefringence for gammadion nanoarrays with varying thicknesses. The scale bar corresponds to 0.05 μm^−^^1^

Corresponding momentum-space (Fourier domain) measurements for different thicknesses are presented in Fig. 4. For each thickness, the full 4 × 4 polarization Mueller matrix is recorded in the Fourier plane, and the key descriptors of chiro-optical effects, namely the CDA (*M*_14_/*M*_41_) and CB (*M*_23_/*M*_32_) elements, are analyzed for comparison. The increase in the magnitude of both the CDA and CB with the sample thickness is clearly visible in Fig. 4a, b. Importantly, even the very weak chiral response of the 50 nm structures is resolved via the Fourier-domain polarimetry approach, which was not the case for the spectral measurements, as shown in the Supplementary information Fig. S1. The chiro-optical response of the 50 nm gammadion structure also exhibits the symmetric and antisymmetric features in the respective elements, consistent with the expected behavior of a chiral medium (the Mueller matrix of the 50 nm thick gammadion structure is presented in the Supplementary Information Section S7, Fig. S7).

The extracted momentum-space chiro-optical maps also carry distinct signatures of the multipolar modes excited within the gammadion structures. For instance, the experimentally observed CB and CDA maps reveal different spatial patterns: while the CB exhibits a relatively homogeneous distribution across the Fourier plane, the CDA appears more structured and concentrated near the center. This momentum-domain distribution contrast can be directly interpreted in terms of the underlying multipolar excitations. The CB arises from the phase difference accumulated between LCP and RCP components. Accordingly, it is predominantly governed by the real part of the induced multipole moments, which tends to vary slowly with angle for resonant modes, resulting into a smoother and more uniform CB distribution in momentum space. On the other hand, CDA originates from the differential amplitude between the opposite circular polarizations. Hence, it is primarily dictated by the interference among the mutipoles, which can be constructive for one handedness and destructive for the other at a specific scattering angle. This angularly selective interference leads to the highly localized and structured CDA observed near the center of the Fourier plane. The presence of higher order multipoles in our structure yields a significant CDA in the forward direction. The evolution of the chiro-optical maps with the thickness of the gammadion structures reflects the changing relative excitation strength of the contributing multipoles. In addition, we have also calculated the mean value of the CB and CDA parameters around the center of the Fourier plane, and the maximum achievable magnitude. These quantitative parameters further characterize the system’s chirality and serve as useful metrics for comparing enantiomers, as shown in the Supplementary Information, Section S8.

The ability of Fourier-domain Mueller matrix polarimetry to sensitively probe weak chiral effects motivates further exploration of this platform for enantiomer characterization, a central application in chiral plasmonics^19,21^.

**Fig. 5:**
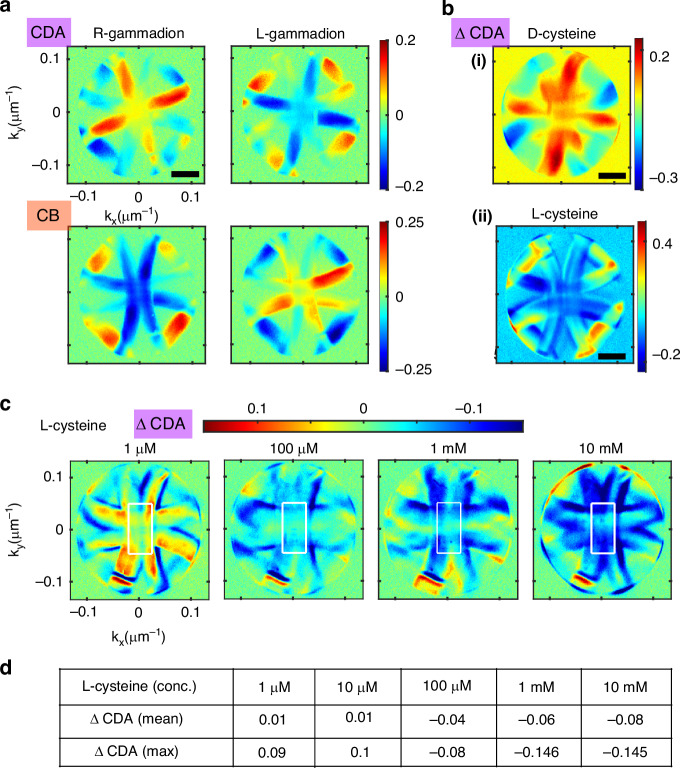
Momentum space polarimetry for characterization of structural enantiomers and label-free detection of chiral molecules. **a** Fourier domain enantiomer characterization of L- and R- type gammadion arrays with 50 nm thickness. Momentum-space maps of both the circular diattenuation and circular birefringence shows a clear sign reversal between the two enantiomeric structures. **b** Molecular enantiomer sensing: the change in circular diattenuation (ΔCDA) due to the presence of L-cysteine (i) and D-cysteine (ii) is used as a metric to distinguish between the molecular enantiomers. **c** Difference between the CDA of the bare and L-cysteine functionalized gammadion arrays (ΔCDA) as a function of cysteine concentration. **d** Mean value of ΔCDA parameter valuated around the center of momentum space (marked with white rectangles) and the maximum achievable ΔCDA, providing an effective metric for concentration-dependent chiral sensitivity measurements. Scale bars in all sub-figures represent 0.05 μm^−^^1^

To validate the effectiveness of our approach to distinguish chiral enantiomers, we fabricate both L- and R-type gammadion arrays (50 nm thick) on the same glass substrate. Due to their minimal intrinsic CDA, these structures serve as ideal test candidates for enantiomer differentiation using Fourier-domain Mueller polarimetry. The momentum (*k*)-space radiation patterns from both bare enantiomers are recorded, and the relevant Mueller matrix elements (CDA and CB descriptors) are presented in Fig. 5a. While subtle spatial inhomogeneities are present in the chiro-optical response, the key observation is that both the CDA and CB maps exhibit reversal in sign between the two enantiomers, effectively forming mirror images of each other. This is a clear indication of enantiomeric contrast.

Notably, the linear birefringence elements in the Mueller matrix remain unchanged for both enantiomers. This highlights certain additional advantages for the utilization of the Mueller matrix. Circular anisotropic effects are sensitive to the structure handedness, whereas linear anisotropy is invariant, since linearly polarized light cannot distinguish between opposite circular polarizations. The Mueller matrix framework provides a natural plateform where the resultant polarization anisotropic effects with circular and linear anisotropic origins are decoupled and reflected in the different elements of the Mueller matrix, thereby enabling unambiguous characterization of structural chirality. This capability is promising for investigating spatial or angular inhomogenous chiral or achiral effects associated with complex nanostructures.

Next, we study the potential of our employed system for sensing molecular enantiomers. Conventional CD spectroscopy leverages the differential interaction of enantiomers with left- and right-circularly polarized light to determine molecular handedness. However, its effectiveness is often limited by the inherently weak chiro-optical responses exhibited by most chiral molecules^9–12^. In recent years, chiral plasmonic nanostructures have emerged as powerful platforms to address this issue by significantly enhancing the sensitivity of the chiro-optical measurements^5,21,22^. These nanostructures possess the ability to generate localized superchiral fields according to the handedness of the structure^23^, which strongly interacts with the attached chiral molecules. When chiral molecules are adsorbed on such platforms, their interaction with these enhanced superchiral near-fields produces distinct optical signatures for opposite enantiomers, thereby enabling sensitive detection and characterization^5,21–23^.

In our study, the far-field polarized radiation carries the imprint of these near-field interactions, making the observed response sensitive to the handedness of the molecules adsorbed on the gammadion array. We selected cysteine, a thiol-containing amino acid, as a model chiral molecule due to its chiral configuration relevance in biology, including protein synthesis^45^, metabolism^46^, neurophysiology^47^, to name a few; and its strong affinity for gold surfaces. In this work, L-cysteine and D-cysteine molecules were adsorbed onto the 150 nm thick gammadion arrays, which exhibit strong intrinsic chiro-optical effects, Fig. 4. Briefly, the gammadion arrays were immersed in 1 mM aqueous solutions of L- or D-cysteine for 2 h, then washed with deionized water and dried in nitrogen. As has been shown in previous studies^48^, thiol-containing cysteine molecules form a strong covalent bond with Au atoms, ensuring a uniform and dense coverage over a complete gammadion surface. Fourier-plane CDA responses are then recorded after functionalization with both L- and D-cysteine. The change in the resultant CDA magnitude relative to the bare (unfunctionalized) gammadion arrays, termed as ΔCDA, serves as the metric for enantiomer discrimination, Fig. 5b. We observe that adsorption of D-cysteine (matching the handedness of the gammadion) enhances the chirality of the overall system, whereas adsorption of L-cysteine reduces it. While the magnitude of the CDA change in momentum space ∣ΔCDA∣ yields similar value for both enantiomers ≈0.2 (as the same 1 mM concentration was used), the sign of the change ∣ΔCDA∣ is opposite as shown in Fig. 5b, thereby enabling the characterization of the attached molecular handedness. We additionally performed control experiments by depositing an analogous achiral molecule onto the gammadion substrate to assess any changes in the CDA maps arising from molecular adsorption or variations in the bulk refractive index. Measurements were carried out for both left- and right-handed gammadion arrays, and the CDA maps were compared before and after functionalization with the achiral molecule. No significant changes were observed in the momentum-resolved CDA maps for either structure, confirming that the observed differential signal in the momentum domain chiro-optical maps arises from chiral molecular interactions with the gammadion arrays. The corresponding results are presented in Supplementary Information Fig. S9.

Furthermore, we validate the performance of the momentum-space polarimeter by probing chiral sensing with varying concentrations of cysteine molecules. For this purpose, several gold gammadion arrays of fixed thickness (150 nm) are fabricated, and a specific enantiomer of cysteine (L-cysteine) is deposited on the structures with different concentrations. Prior to molecular functionalization, the intrinsic chiro-optical response of the bare gammadion arrays is quantified. Momentum-space CDA responses are then recorded after functionalization, and the difference between the CDA of the bare and cysteine-coated gammadion arrays is used as the primary sensing metric. The corresponding results are shown in Fig. 5c, d. No appreciable change in the magnitude of the momentum-space CDA is observed for cysteine concentrations up to 100 μM. The obtained magnitude of ΔCDA is shown for 1 μM, 10 μM concentrations of the cysteine, while only the spatial maps corresponding to 1 μM are shown here. Beyond this threshold, the CDA difference increases with concentration. The cysteine concentration is varied gradually up to 10 mM, and results corresponding to specific cysteine concentrations are shown. In Fig. 5d, we present both the mean CDA value evaluated near the center of momentum space and the maximum achievable CDA magnitude, which together provide an efficient metric for concentration-dependent chiral sensitivity measurements.

## Discussion

In summary, we have demonstrated a Fourier-domain Mueller matrix approach for probing the chiro-optical response of nanoplasmonic arrays. By integrating dark-field Fourier imaging with full Stokes and Mueller matrix formalism, we were able to capture polarization-resolved radiation patterns that reflect both intrinsic structural chirality and the excited electromagnetic modes within the structures. We also investigated the dependence of chiro-optical responses on the thickness of the nanostructures. The Mueller matrix formalism proved effective not only for simultaneously capturing circular birefringence (CB) and circular diattenuation (CDA), but also for decoupling linear and circular anisotropic effects. Furthermore, we demonstrated that the combination of plasmonic gammadion structures with Fourier-domain polarization measurements provides a sensitive platform for distinguishing molecular enantiomers through their influence on polarization-resolved far-field scattering. These findings establish Fourier-domain Mueller polarimetry as a powerful and versatile technique for characterizing chiral nanostructures and enabling label-free enantiomer detection.

## Methods

### Dark-field Fourier-domain polarimetry setup

The chiro-optical response of the gammadion nano-arrays is characterized using a custom-built dark-field Fourier-domain Mueller matrix imaging platform integrated into a commercial inverted microscope (Olympus IX73). The microscope’s built-in halogen illumination source (100 W) provides broadband excitation, which is first collimated and then passed through a rotatable achromatic linear polarizer (wire-grid, 400−700 nm) followed by an achromatic quarter-wave plate (350−850 nm), enabling the generation of arbitrary incident polarization states. A dark-field condenser (NA ≈ 0.7) focuses the light into an annular distribution at the sample plane, ensuring that only the scattered light from the gammadion arrays is collected by a long-working-distance objective (Olympus MPlanFL, NA = 0.5), thereby eliminating direct illumination. The back focal plane of the collection objective—containing the angular scattering distribution—is relayed onto the entrance plane of the spectrometer camera (Andor Kymera spectrograph with Newton EM-CCD) using a lens system designed to preserve the Fourier-plane geometry. Polarization-resolved detection is implemented using another pair of rotatable achromatic quarter-wave plates and linear polarizer (identical specifications as in the illumination arm) placed in the detection path. By sequentially generating and analyzing a complete set of linear and circular polarization states, full Fourier-plane Mueller matrices are acquired. All polarization optics are mounted on computer-controlled motorized rotation stages (PRM1/M-Z7E, Thorlabs, USA), providing high angular accuracy and fully automated measurement sequencing. The polarizers and wave plates are carefully aligned perpendicular to the beam propagation direction to avoid tilt-induced polarization distortions. Spectral selection is performed using narrow band-pass filters (BP 649–709 nm). The detailed polarization algebra and computational steps used to construct the Mueller matrix are provided in Supplementary Information (Section S4).

### Plasmonic gammadion structure

**Fig. 6:**
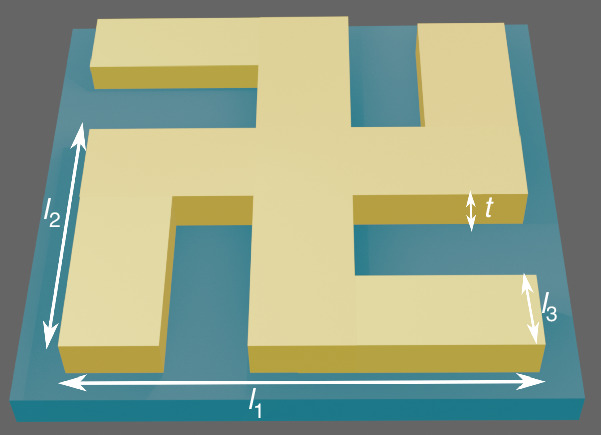
Schematic illustration of the geometrical parameters of the gold gammadion nanoarrays (periodicity, arm lengths (*l*_1_, *l*_2_, *l*_3_), and thickness (*t*)). *l*_1_ = 400 nm, *l*_2_ = 240 nm, *l*_3_ = 80 nm, and *t* is varied for 50 nm, 100 nm, 150 nm

The geometrical parameters of the gold gammadion nanoarrays (periodicity, arm length, and thickness) were optimized via numerical simulations, and are schematically illustrated in Fig. 6. The nanostructure arrays are composed of gold gammadion structures with a periodicity of 800 nm in both *x* and *y* directions, on a thin Glass substrate (145 μm thick D263T wafer, 4 in). The Au layer is characterized by a thickness of 50, 100, and 150 nm for three different samples using electron beam evaporator (Alliance-Concept EVA 760, pressure 8.0*E* − 07 mbar); a 2 nm titanium layer is added between the Au layer and the substrate for adhesion. On top of the Au layer, 40 nm thick chromium layer is deposited as a hard mask. The nanostructures are fabricated by means of electron beam lithography (Raith EBPG5000+ tool) using a negative resist (HSQ 6%, DuPont, XR-1541-006). The chromium layer and the remaining resist are eliminated using a series of etching procedures, including ion beam etching (Veeco Nexus IBE 350, Low IBE) and chemical etching by Cr etch (a mixture of cerium ammonium nitride and perchloric acid). The images showing the appearance and the dimensions of the resulting samples are obtained using atomic force microscopy analysis (Bruker FastScan) and the scanning electron microscopy (Zeiss SEM Crossbeam 550, SE Detector).

Full-wave numerical simulations were carried out using the commercially available finite-element electromagnetic solver COMSOL Multiphysics (Wave Optics Module). Floquet periodic boundary conditions were applied at the lateral boundaries of the simulation domain. Illumination was introduced through a periodic excitation port in the substrate (glass) region, while the polarization state of the transmitted field was analyzed using a receiver port positioned in the superstrate (air) region. The frequency-dependent electric permittivity of gold was modeled using interpolated experimental data^49^, and the substrate was assumed to have a constant refractive index of (*n* = 1.5). Multipole decomposition was performed following the approach described in ref. ^50^, using the near-field data extracted from the full-wave simulations.

## Supplementary information

Supplementary information for: Decoding chirality at the nanoscale with momentum-space polarimetry

## Data Availability

The data and algorithms utilized to produce the results reported in this study are available from the corresponding authors upon reasonable request.
