# Supplementary material for: Decoding chirality at the nanoscale with momentum-space polarimetry
Source: Light Sci Appl. 2026 May 18;15:235. doi: 10.1038/s41377-026-02336-z (PMC13181049; doi:10.1038/s41377-026-02336-z)
Supplement: Supplementary file 1 — Supplementary information for: Decoding chirality at the nanoscale with momentum-space polarimetry [file 41377_2026_2336_MOESM1_ESM.pdf]

Supplementary information for : Decoding chirality at the nanoscale with momentum-space polarimetry

Jeeban Kumar Nayak*[1], Meghna Sarkar[1], Siarhei Zavatski[1], Ebru Buhara[1], Sergejs Boroviks[1], and Olivier J. F. Martin†[1]

[1]Nanophotonics and Metrology Laboratory (NAM), Swiss Federal Institute of Technology Lausanne (EPFL), Lausanne 1015, Switzerland

S1: Spectral variation of the circular dichroism effect exhibited by the gammadion arrays

The chiro-optical response of the nanostructures is typically characterized by their circular dichroism (CD) spectra. Figure S1 presents both experimental (Fig. S1(a)) and numerical (Fig. S1(b)) CD spectra of gammadion arrays with different thicknesses. As per design, the arrays exhibit pronounced chirality within the 600–700 nm wavelength range. A decrease in the thickness of the gammadion structures reduces the CD magnitude, demonstrating the strong dependence of the chiro-optical response on the geometrical parameters. For 50 nm thick gammadions, a very weak or negligible amount of CD is observed across the spectral range. The experimental CD spectra follow the same trend, with the 50 nm-thick arrays showing a response similar to that of the bare substrate. A red shift and resonance broadening is observed experimentally compared to the simulations, which can be attributed to fabrication imperfections and deviations between the ideal design parameters and the real fabricated structures.

*jeeban.nayak@epfl.ch
†olivier.martin@epfl.ch

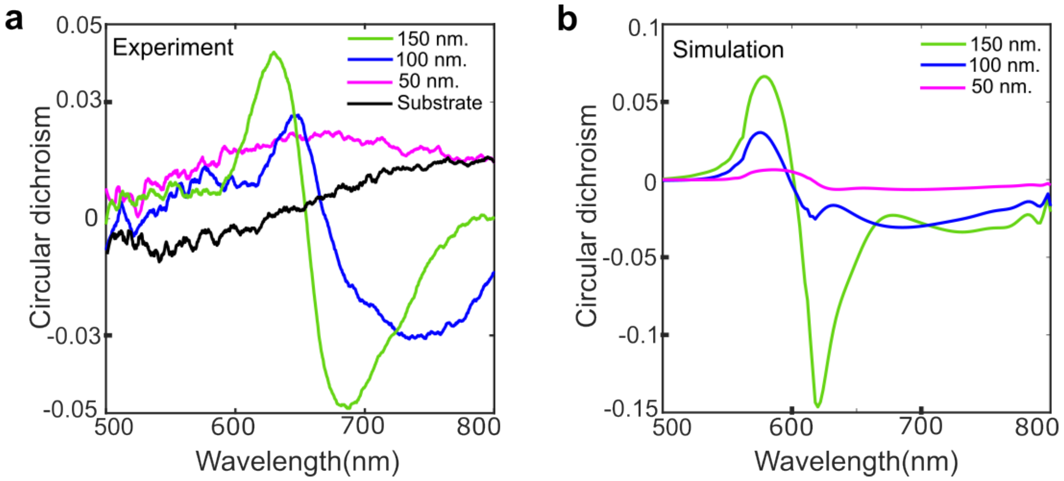

Figure S1: (a) Experimental and (b) simulated CD spectra of gammadion arrays with varying thicknesses."

S2: Apperance of arc-like intensity patterns in the Fourier plane

In the high numerical aperture (NA) dark-field configuration, the sample is illuminated by an azimuthally varying distribution of oblique incidence angles. Because the NA of the collection objective is smaller than that of the dark-field condenser, the zeroth-order (direct) beam is excluded from detection. The diffraction angle is determined by the illumination wavelength and the periodicity of the gammadion arrays. Under our experimental conditions, the finite NA of the collection objective allows detection primarily of the -1 diffraction orders, while higher diffraction orders become evanescent and therefore do not contribute to the measured signal. Schematic illustrations of the resulting arc-like intensity patterns in the Fourier plane are shown in Fig. S2(a,b).

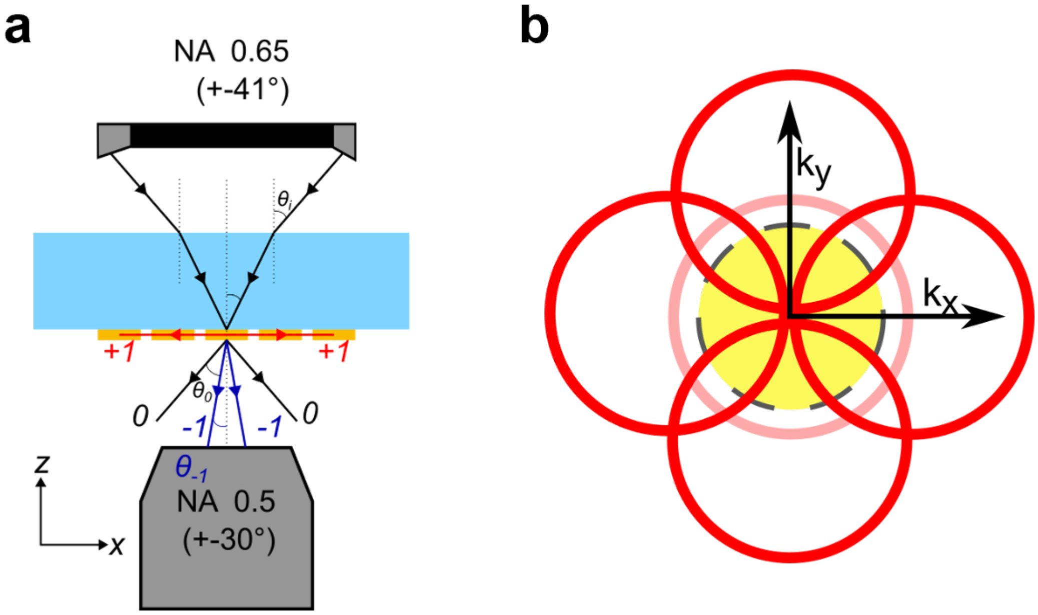

Figure S2: Schematic illustration of diffraction-order collection from the periodic gammadion arrays (a) and the resulting origin of the arc-like intensity patterns in the Fourier plane. The numerical-aperture region of the collection objective is highlighted in yellow.

S3: Multipole decomposition

The contribution of different electromagnetic modes to the chiro-optical response of the gammadion structure is analyzed through multipole decomposition of its scattering response. The expansion includes both electric and magnetic multipoles up to the octupole order. The strongest contributions arise from the magnetic quadrupole and electric octupole modes. The coupling between these higher-order modes produces bonding and antibonding features for LCP and RCP excitations, respectively, leading to distinct scattering responses that manifest as the observed CD.

S4: Manifestation of the chiro-optical responses in the polarization Mueller matrix

The Mueller matrix in the Fourier plane is constructed from 36 polarization-projection measurements. This overdetermined basis reduces measurement errors and enables a robust physical interpretation. The chiro-optical responses manifest in specific Mueller matrix elements, which are decoupled from the effects of linear anisotropy. For instance, the M_{14} element can be expressed as

$$M_{14} = RR + RL - LL - LR = R(R + L) - L(L + R) = RI_{total} - LI_{total} \tag{1}$$

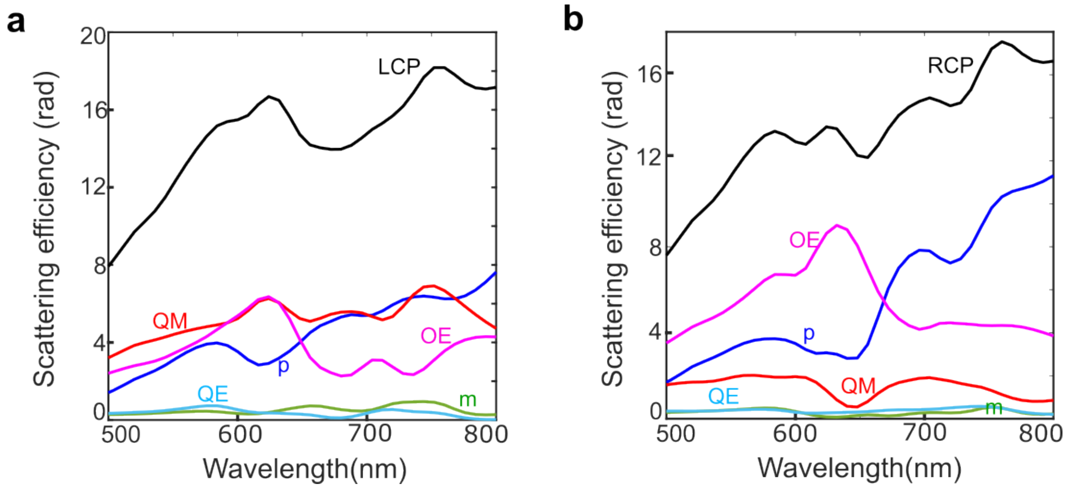

Figure S3: Multipole decomposition of the modes excited in a gammadion structure under (a) left- and (b) right-circularly polarized illuminations. p, m: electric and magnetic dipoles; QE, QM: electric and magnetic quadrupoles; OE: electric octupole.

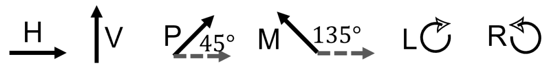

HH+HV+VH+VV	HH+HV-VH-VV	PH+PV-MH-MV	RR+RL-LL-LR
HH-HV+VH-VV	HH-HV-VH-VV	PH-PV-MH+MV	RH-RV-LH+LV
HP+VP-HM-VM	HP-VP-HM+VM	PP-PM-MP+MM	RP-RM-LP+LM
RR+LR-RL-LL	HR-VR-HL-VL	PR-PL-MR+ML	RR-RL-LR+LL

Figure S4: Construction scheme for the polarization Mueller matrix using 36 polarization-projective measurements. Input (first letter) and analyzer (second letter) polarization states: $H(horizontal)$, $V(vertical)$, $P(+45\,\mathrm{deg})$, $M(135\,\mathrm{deg})$, L left circular polarized (LCP), R right circular polarized (RCP)

Thus, M_{14} describes the difference in total scattering between RCP and LCP excitations. When normalized by M_{11} (the total scattered intensity), this corresponds to the standard asymmetry parameter g, commonly used to quantify the chiro-optical response, and here refered as circular diattenuation (CDA). Now let us consider the symmetry exhibited by different Mueller matrix elements in the case of a chiral medium. As discussed in the main text, the M_{14} and M_{41} elements are symmetric, i.e., they are expected to display similar behaviors. The origin of this symmetry can be understood from the construction of the corresponding Mueller matrix elements. A difference between them would only arise if there were an asymmetric conversion between the circular components $(LR - RL)$. However, since the difference between the parallel circularly polarized components is identical for both elements, and because neither CDA nor CB contributes to cross-conversion between opposite circular polarizations, M_{14} and M_{41} remain equal in a typical chiral medium. Using similar algebraic interpretation contribution from the extrinsic chirality can also be obtained.

In contrast, the optical rotation effect, associated with circular birefringence (CB), manifests as an anti-symmetric relation $M_{23} = -M_{32}$. This arises because CB induces the rotation of an incident linearly polarized beam. Both M_{23} and M_{32} are specifically constructed to measure this rotation. For example, a chiral medium rotates the polarization angle of an incident horizontally or vertically polarized beam, and the resulting state can be analyzed by projecting onto orthogonal linear polarization bases. Conceptually, this corresponds to determining the rotation of a vector by evaluating its orthogonal components.

S5: Calibration of the dark-field Mueller matrix setup

Calibrating the Mueller matrix polarimeter is essential to ensure accurate measurement of the polarization properties of a sample. In principle, measurement errors can originate from each optical element used in the experimental setup. Therefore, it is important to determine the true polarization behavior of the system and compensate for its non-ideal polarization responses. In this work, we employ a well-established eigenvalue calibration method (EVC) to spectrally calibrate the dark-field Mueller matrix arrangement. Details of the calibration procedure can be found in earlier works [1–3]. The calibration samples consist of two different orientations of the polarizers (28° and 73°) and the quarter-wave plate (23° and 68°), which are known to be optimal for eigenvalue calibration. Using this method, we demonstrate that it is possible to retrieve the true polarization properties of a sample in a dark-field Mueller matrix measurement.

To illustrate this, we first present the raw Mueller matrix of the system without any structure—often referred to as the blank Mueller matrix—which ideally corresponds to the identity matrix (Fig. S5(a)). The elements exhibiting significant deviations from this ideal behavior are the M_{34} and M_{43} components. These terms indicate the presence of a linear retarder with a 45° fast-axis orientation. This unwanted polarization response arises from the polarization-sensitive beam splitter integrated into the Olympus microscope system. However, these non-ideal behaviors are effectively compensated using the eigenvalue calibration method, as shown in the calibrated Mueller matrix in Fig. S5(b). The calibrated blank Mueller matrix approaches an identity matrix, with all off-diagonal elements near zero, including the previously problematic M_{34} and M_{43} elements. Furthermore, we present the calibrated Mueller matrix of a quarter-wave plate with a 23° orientation Fig. S5(c). The extracted polarization parameters serve as quantitative metrics for validating the system's performance. As expected, the linear retardance approaches $\pi/2$ (Fig. S5(d)), while the diattenuation parameter remains near zero (Fig. S5(e)), demonstrating the capability of the Mueller matrix polarimeter to accurately quantify the polarization properties of a sample.

S6: Intrinsic Mueller matrix of the system in the momentum-space

In an ideal dark-field configuration, no light should be collected in the absence of a scatterer or sample. Furthermore, typical polarimetric calibration elements—such as polarizers and retarders—are largely transparent and therefore do not provide detectable scattering under dark-field illumination. To record the intrinsic Mueller matrix of the system, we therefore used a bare glass substrate with negligible polarization anisotropy. This allowed a small amount of scattered light to reach the detector. Nevertheless, the collected signal was insufficient for a complete Mueller matrix reconstruction, requiring us to slightly adjust the position of the collection objective to intercept more of the illumination. The corresponding Mueller matrix is presented in Fig. S6(a).

Although the resulting Mueller matrix resembles an identity matrix with diagonal elements close to unity, several off-diagonal elements exhibit non-negligible magnitude with distinct inhomogeneous spatial variations. These features warrant detailed discussion. The elements $M_{12/21}$, and $M_{13/31}$ elements exhibit $\cos 2\phi$, and $\sin 2\phi$, lobe like patterns respectively. Similar azimuthal pattern with weak magnitude also appera in the $M_{34/43}$, and $M_{24/42}$ elements. The $M_{12/21}$, and $M_{13/31}$ elements represents linear diattenuation, in this case, we observe they describe the presence of an azimuthally varying effective linear diattenuator with azimuthally varying orientation axis. Such polarization conversion originates from the high NA illumination/collection and the associated spin–orbit interactions that arise in tightly focused fields [4–6]. When the system is further displaced from the ideal dark-field condition, so that a substantial portion of the annular illumination is directly collected—the effect becomes more pronounced. The Mueller matrix recorded in this scenario, shown in Fig. S6(b), corresponds to a diattenuating retarder with an azimuthally varying fast-axis orientation. While tight focusing is known to induce finite linear diattenuation, it does not produce significant retardance [4;7]. Therefore, the presence of non-zero retardance terms in Fig. S6(b), combined with the spectral Mueller matrix observations from Fig. S5(a), indicates the presence of an additional polarization-sensitive optical component in the detection pathway, the beam splitter present in the microscope.

Importantly, the M_{23}, and M_{32} elements in Fig. S6(b) share the same sign, a characteristic signature of linear polarization anisotropy. In contrast, chiral media produce M_{23}, and M_{32} of of opposite signs describing

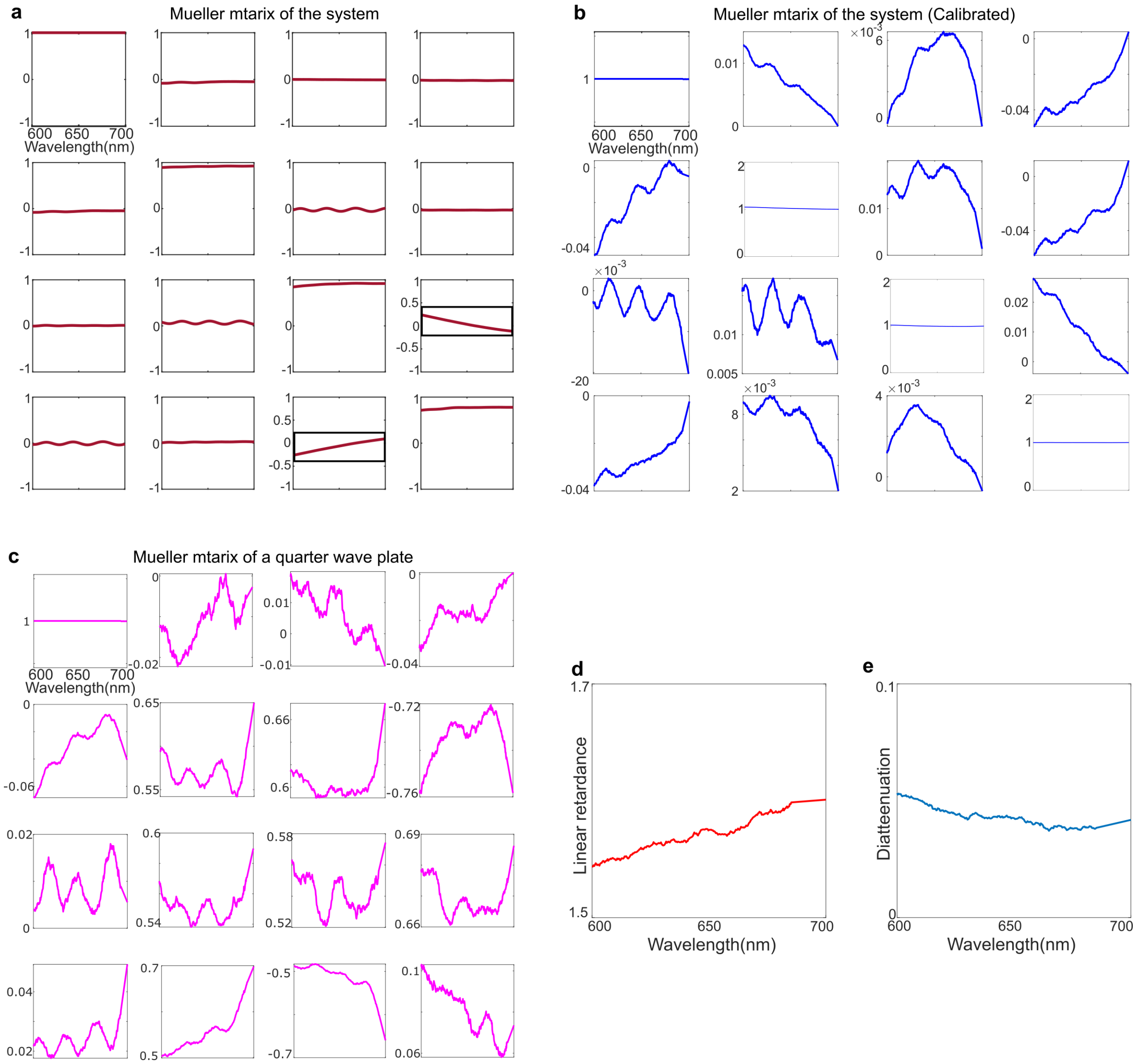

Figure S5: Spectral-domain eigenvalue calibration of the Mueller matrix in the dark-field arrangement. (a) Raw Mueller matrix of the system without any sample (blank Mueller matrix). The elements showing significant deviation from the identity matrix, M_{34} and M_{43}, are highlighted with black rectangular boxes. (b) Mueller matrix of the system after applying the eigenvalue calibration method, accurately reproducing an identity matrix with all off-diagonal elements approaching zero. (c) Calibrated Mueller matrix of a quarter-wave plate with a 23° orientation axis. (d) Extracted linear retardance parameter approaches the expected value of $\pi/2$, and the linear diattenuation parameter (e) remains close to zero.

polarization rotation, as observed in the case of the gammadion arrays. Thus, the present results not only disentangle the origins of individual system-induced Mueller matrix elements but also confirm that the Mueller matrices measured for the chiral gammadion samples capture the genuine chiro-optical response of the structures.

S7: Momentum domain Mueller matrix of the 50 nm thick gammadion structures

Figure S7 shows the complete 4×4 momentum-domain Mueller matrix for the 50 nm-thick gammadion arrays. Despite their weak chiro-optical response, the polarization Mueller matrix successfully probes and quantifies the residual effects. Both the circular diattenuation (M_{14}, M_{41}) and circular birefringence (M_{23}, M_{32}) elements display the characteristic symmetric and antisymmetric behavior of a chiral medium, consistent with thicker gammadion arrays albeit with reduced magnitude. By contrast, elements corresponding to linear anisotropy (e.g., linear birefringence M_{24}, M_{42}, M_{34} and M_{43}) do not show significant variation. This validates the ability of the Mueller matrix to decouple linear and circular anisotropy, providing an efficient platform for probing, quantifying, and interpreting chiro-optical responses in arbitrary chiral media.

S8: Statistical metrics of circular birefringence and circular diattenuation in the momentum domain

The momentum-space distributions of circular diattenuation and circular birefringence for the gammadion arrays were obtained from the full $k-$ domain Mueller matrix measurements. Both parameters exhibit inherently inhomogeneous spatial patterns, with their maximum magnitudes primarily ocorresponds to the center of the Fourier plane ($k_x, k_y \approx 0$). While the circular diattenuation displays a more structured and angularly localized response, the circular birefringence shows a comparatively smoother spatial variation, reflecting the distinct physical origins of amplitude- and phase-based chiro-optical effects. Here, we report the mean and maximum values of these chiro-optical parameters for gammadion arrays of different thicknesses and handedness. These quantitative metrics not only provide a measure of the overall chiral strength of the system but also offer a useful basis for distinguishing between enantiomeric geometries.

S9: Chiro-optical dispersion maps from the gammadion arrays.

Wavelength/frequency resolved measurements combined with momentum-space imaging can provide additional insight into the chiro-optical response of the gammadion arrays. Here we present representative dispersion maps capturing the combined spectral and momentum-space behavior of the structures. The dispersion maps reveal an enhanced magnitude of the CDA parameter around $k_y = 0$, consistent with the momentum-space CDA distributions discussed in the main text. In addition, the wavelength–momentum representation uncovers multiple spectrally and momentum-selective features, indicative of underlying photonic and plasmonic resonances supported by the gammadion arrays. A comprehensive analysis of the origin and coupling mechanisms of these dispersive features lies beyond the scope of the present work and will be addressed in a dedicated future study. Here, the dispersion maps are included to demonstrate that the momentum-space polarimetric platform is capable of simultaneously resolving chiro-optical responses in both spectral and reciprocal-space domains.

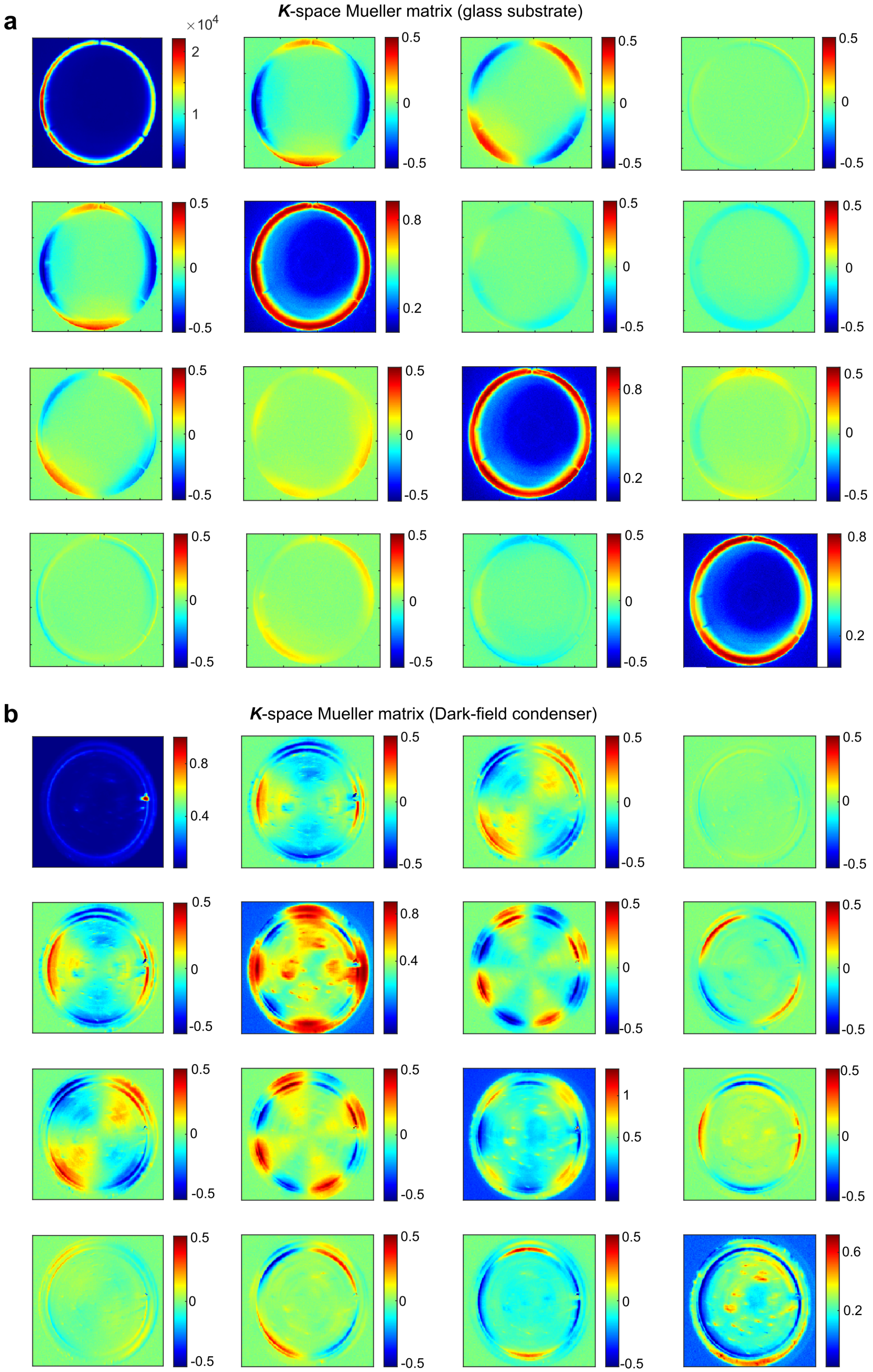

Figure S6: (a) Intrinsic momentum-space ($k-$ space) Mueller matrix of the system obtained using a bare glass substrate. (b) Corresponding Mueller matrix highlighting the polarization conversion introduced by the dark-field condenser when the collection objective is displaced to capture the annular illumination.

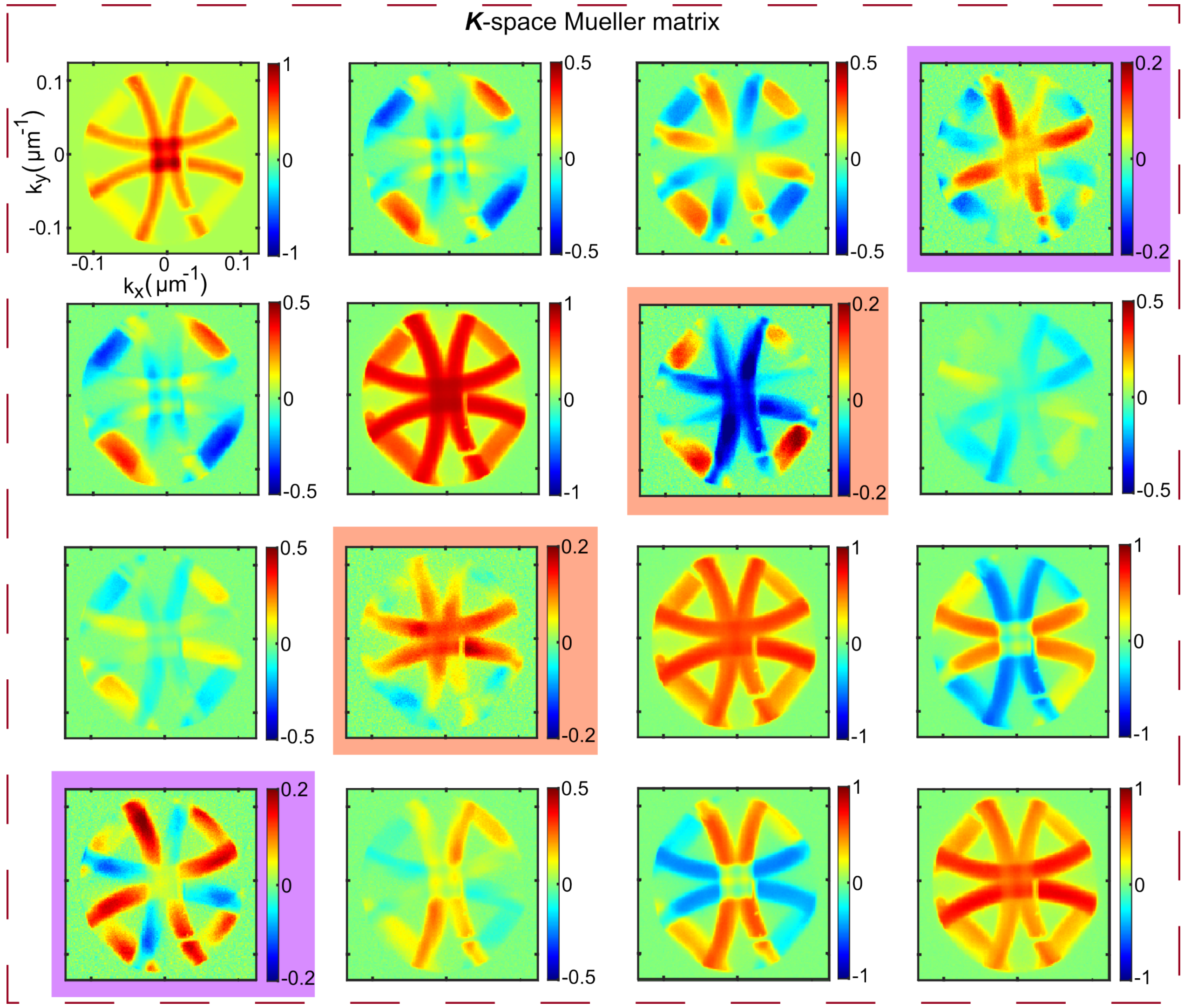

Figure S7: Fourier-domain polarization Mueller matrix of 50 nm-thick gammadion arrays. Circular anisotropy descriptor elements M_{14}, M_{41} (CDA) and M_{23}, M_{32} (CB) are highlighted.

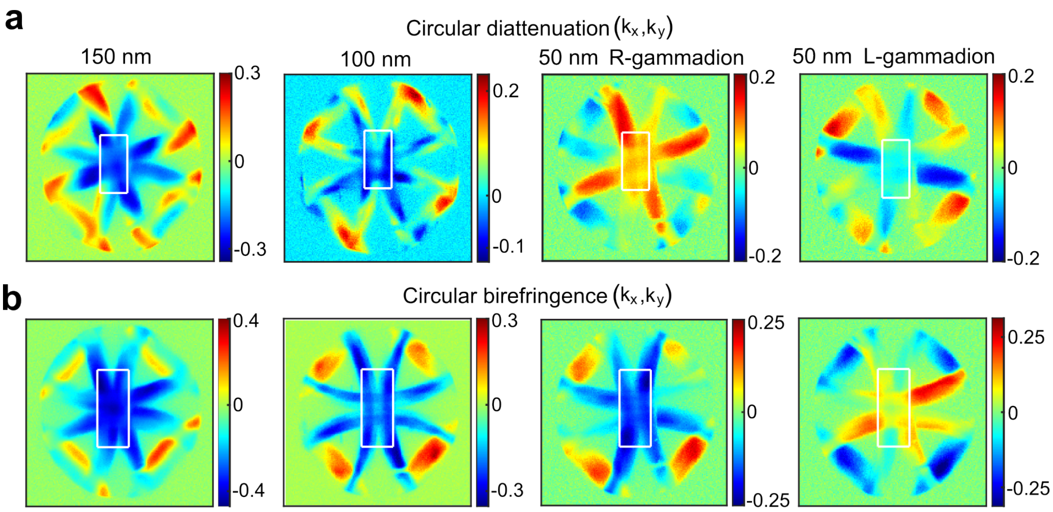

c

	150 nm	100 nm	50 nm (R)	50 nm (L)
CD(Mean)	-0.19	-0.04	0.07	-0.03
CD(Max)	-0.34	-0.13	0.16	-0.17
CB(Mean)	-0.31	-0.20	-0.15	0.03
CB(Max)	-0.44	-0.35	-0.25	0.21

Figure S8: Mean and Peak Values of Fourier-Plane Chiro-Optical Parameters. Momentum-domain spatial distributions of the circular diattenuation (a) and circular birefringence (b) parameters are shown for gammadion arrays with varying thicknesses and opposite handedness. The mean values reported in table (c) are calculated over the region indicated by the white rectangle in each corresponding map, while the peak values are extracted from the full spatial distributions.

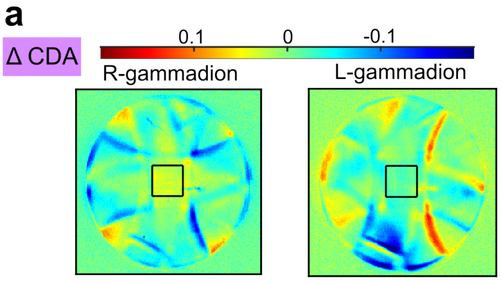

b

Achiral Molecule	R-gammadion	L-gammadion
ΔCDA (mean)	0.02	-0.01
ΔCDA (max)	0.04	0.02

Figure S9: (a) An achiral molecule cysteamine is deposited onto gammadion arrays from 1 mM aqueous solution of cysteamine hydrochloride salt using the same procedure as described in the main text. Apart from the achiral nature of cysteamine, the choice of the molecule was also governed by its similarity with L- and D-cysteine in molecular weight and availability of free thiol group, which promotes adsorption onto gold surfaces. The corresponding changes in the momentum-domain circular diattenuation (ΔCDA) maps before and after functionalization of gammadions with opposite handedness are presented. (b) The mean and maximum values in the central region of the maps are summarized for quantitative comparison. No significant changes are observed in the momentum-resolved CDA maps for either structure. This result confirms that non-specific adsorption or bulk refractive index changes do not produce measurable CDA variations. Therefore, the observed differential signal presented in Fig. 5 of the main text primarily arises from chiral molecular interactions with the gammadion arrays.

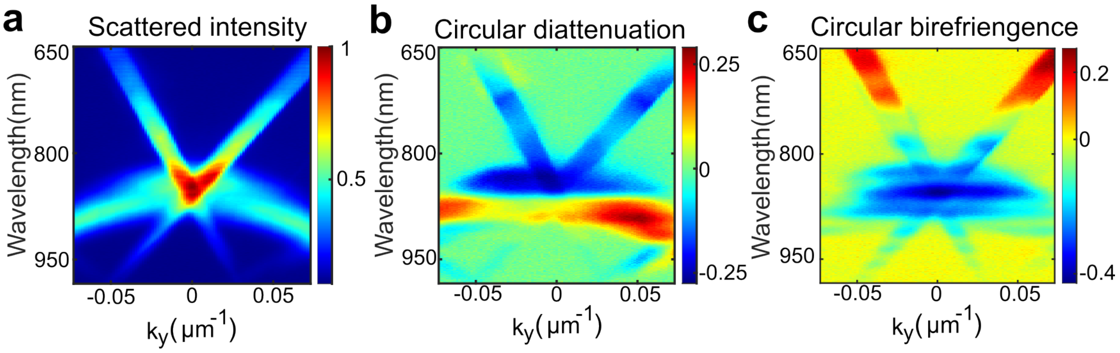

Figure S10: Dispersion maps obtained from 150 nm thick gammadion arrays showing the variation of (a) total scattered intensity, (b) circular diattenuation, and (c) circular diattenuation.

References

[1] Antonello De Martino, Enric Garcia-Caurel, Blandine Laude, and Bernard Drévillon. General methods for optimized design and calibration of mueller polarimeters. *Thin Solid Films*, 455:112–119, 2004.

[2] Blandine Laude-Boulesteix, Antonello De Martino, Bernard Drévillon, and Laurent Schwartz. Mueller polarimetric imaging system with liquid crystals. *Applied optics*, 43(14):2824–2832, 2004.

[3] Jalpa Soni, Harsh Purwar, Harshit Lakhotia, Shubham Chandel, Chitram Banerjee, Uday Kumar, and Nirmalya Ghosh. Quantitative fluorescence and elastic scattering tissue polarimetry using an eigenvalue calibrated spectroscopic mueller matrix system. *Optics express*, 21(13):15475–15489, 2013.

[4] Konstantin Y Bliokh, Elena A Ostrovskaya, Miguel A Alonso, Oscar G Rodríguez-Herrera, David Lara, and Chris Dainty. Spin-to-orbital angular momentum conversion in focusing, scattering, and imaging systems. *Optics express*, 19(27):26132–26149, 2011.

[5] Konstantin Yu Bliokh, Francisco J Rodríguez-Fortuño, Franco Nori, and Anatoly V Zayats. Spin–orbit interactions of light. *Nature Photonics*, 9(12):796–808, 2015.

[6] Klas Lindfors, Arri Priimagi, Tero Setälä, Andriy Shevchenko, Ari T Friberg, and Matti Kaivola. Local polarization of tightly focused unpolarized light. *Nature Photonics*, 1(4):228–231, 2007.

[7] Subhasish Dutta Gupta, Nirmalya Ghosh, and Ayan Banerjee. *Wave optics: Basic concepts and contemporary trends*. CRC Press, 2015.
